# A fast magnetic vector characterization method for quasi two-dimensional systems and heterostructures

**DOI:** 10.1038/s41598-023-36803-z

**Published:** 2023-06-14

**Authors:** A. E. Herguedas-Alonso, L. Aballe, J. Fullerton, M. Vélez, J. I. Martín, A. Sorrentino, E. Pereiro, S. Ferrer, C. Quirós, A. Hierro-Rodriguez

**Affiliations:** 1 Departamento de Física, Universidad de Oviedo, 33007 Oviedo, Spain; 2 ALBA Synchrotron, 08290 Cerdanyola del Vallès, Spain; 3 SUPA, School of Physics and Astronomy, University of Glasgow, Glasgow, UK; 4 CINN (CSIC-Universidad de Oviedo), 33940 El Entrego, Spain

**Keywords:** Microscopy, Magnetic properties and materials, Ferromagnetism, Spintronics

## Abstract

The use of magnetic vector tomography/laminography has opened a 3D experimental window to access the magnetization at the nanoscale. These methods exploit the dependence of the magnetic contrast in transmission to recover its 3D configuration. However, hundreds of different angular projections are required leading to large measurement times. Here we present a fast method to dramatically reduce the experiment time specific for quasi two-dimensional magnetic systems. The algorithm uses the Beer-Lambert equation in the framework of X-ray transmission microscopy to obtain the 3D magnetic configuration of the sample. It has been demonstrated in permalloy microstructures, reconstructing the magnetization vector field with a reduced number of angular projections obtaining quantitative results. The throughput of the methodology is × 10–× 100 times faster than conventional magnetic vector tomography, making this characterization method of general interest for the community.

## Introduction

The capability to observe the 3D magnetization vector field at the nanoscale within magnetic systems is of paramount importance for the whole magnetism community. The full comprehension of the physical phenomena arising from the magnetic properties of materials, can be better achieved if there is an experimental window directly showing the three-dimensional magnetization configuration. This access to the magnetization at the nanoscale can be realized using different methods based on acquiring magnetization sensitive images in transmission microscopy setups. For instance, by using Lorentz transmission electron microscopy (TEM)^1–3^, off axis electron holography^4,5^ or differential phase contrast in scanning TEM^6,7^, it is possible to perform vector tomography experiments being able to reconstruct the magnetic induction and the magnetization from arbitrary complex systems^8–10^. X-rays can also be used to access the magnetization configuration at the nanoscale with element specificity. The exploitation of X-ray Magnetic Circular Dichroism (XMCD) in hard and soft X-ray regimes has shown the extreme potential of vector tomography for the observation of magnetic textures and singularities in magnetic microstructures^11,12^, nanostructures^13^ and continuous films^14–16^. This new paradigm of experimental three-dimensional characterization of the magnetization is not restricted to the study of static configurations but permits analyzing the magnetization dynamics in three-dimensions^17^. It also allows to apply the full power of vector analysis to study the topology of magnetic textures^14,18^ giving a different perspective to understand their role in the global framework of the magnetization configuration^19^.

The key element to obtain three-dimensional vector sensitivity is based on the acquisition of differently oriented magnetic images, providing complementary information about the magnetization and its location in space^20–22^. In fact, the volume resolution of the resulting tomogram is directly related with the number of different angular projections as well as their angular range^23^. This makes the experimental acquisition time one of the major limitations of the vector tomography methodology as is common to require up to 2 days to record a complete dataset (~ 10^2^ angular projections). However, when the thickness of the sample is below the axial resolution of the technique, the dimensionality of the reconstruction can be reduced, with high impact on the experimental time. This case is of special interest for the Spintronics, Nanomagnetism and 2D-van der Waals magnetism communities where the quasi-2D geometrical condition is met.

Here we present a fast methodology that can be applied to these quasi-2D magnetic systems for the three-dimensional vector characterization of continuous and patterned magnetic heterostructures. The approach is demonstrated in several rectangular 40 nm thick permalloy (Py, Ni_80_Fe_20_) microstructures with different three-dimensional magnetic configurations. Exploiting the restriction for the magnetization to be contained within a two-dimensional geometry, the reconstruction volume dimensionality is reduced from three to two dimensions, dramatically decreasing the number of angular projections required for the reconstruction. The measurements have been taken at the full-field X-ray transmission microscope (TXM) of the MISTRAL beamline at the ALBA synchrotron^24^. Three-dimensional magnetic states present in the Py microstructures have been resolved showing three different closure domain configurations: two Landau patterns, one mediated by a vortex and another by a cross-tie configuration, and a diamond state. Moreover, we have studied the performance of the method with reduced datasets concluding that with only 6 different angular projections it is possible to obtain qualitative magnetic information with total acquisition times of around 15 min. The presented method allows to obtain speed-ups in experimental acquisition times for quasi-2D systems of × 10- × 100 when compared with conventional magnetic vector tomography.

## Experiment

**Figure 1:**
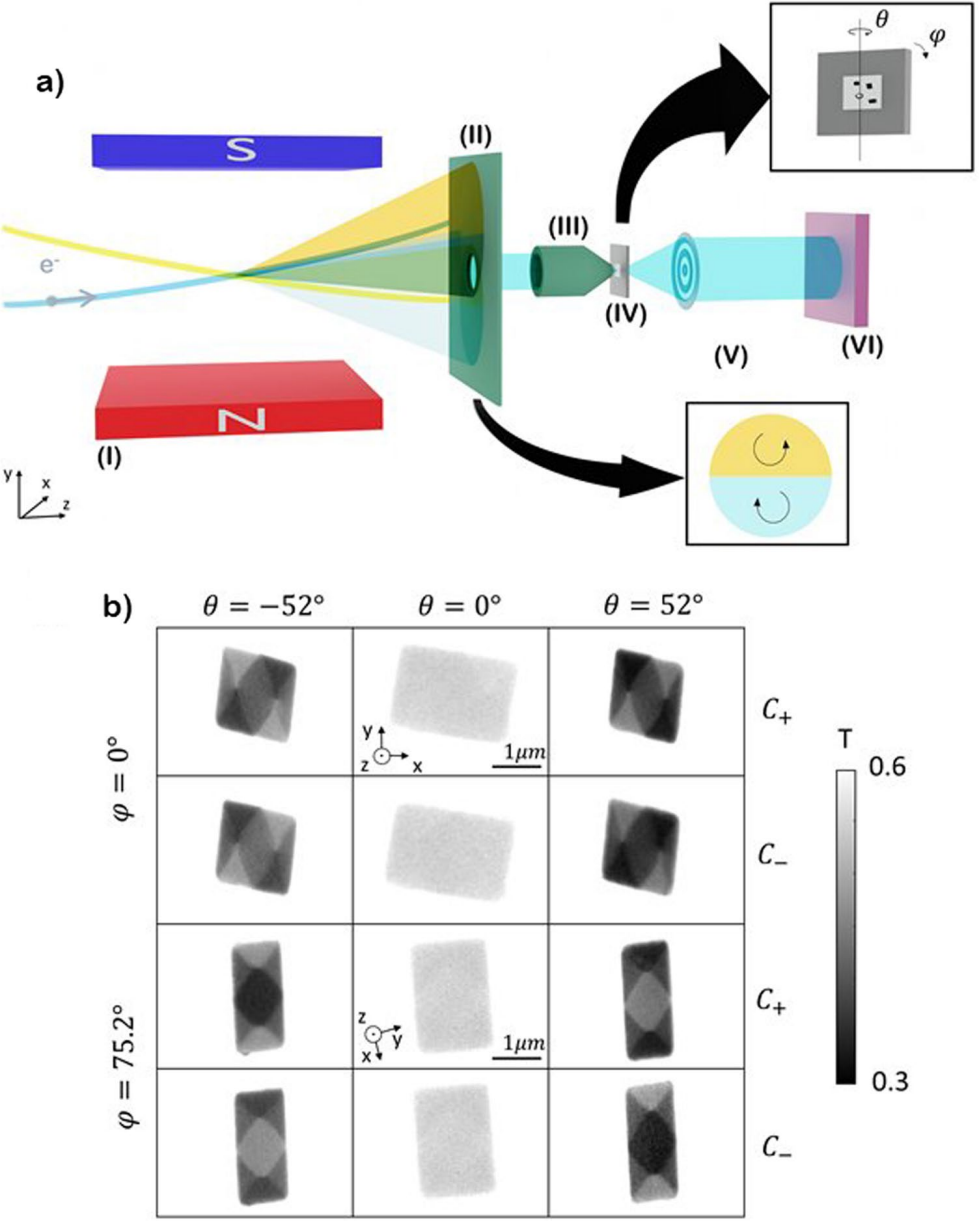
(**a**) Schematic of transmission X-ray microscope, (I) bending magnet, (II) aperture mimicking entrance pupil of the microscope, (III) X-ray condenser, (IV) sample, (V) Fresnel zone plate, (VI) detector. Between (II) and (III) the beamline optics, which work to select the energy and prepare the X-ray beam for the first lens of the microscope, are omitted for clarity. (**b**) Transmittance images for both tilt series with different tilt-angles − 52°, 0°, 52° for circular right (${C}_{+}$) and left (${C}_{-}$) polarizations.

Different magnetic microstructures of 40 nm thick Py were fabricated by conventional e-beam lithography and DC magnetron sputtering on top of Si_3_N_4_ membranes (Ted Pella, 21501-10) for X-ray transmission microscopy experiments. The samples were mounted in the MISTRAL full-field TXM at the ALBA synchrotron^24^. A scheme of the microscope layout is depicted in Fig. 1a. Different circular polarizations of X-rays are obtained in the beamline by properly deflecting within the bending magnet the electron beam present in the storage ring of the synchrotron^25^. In this way it is possible to select if the photons shining the sample come from the top/bottom parts of the emission cone. The transmitted photons form a full field image that is projected on a CCD camera after being magnified by a Fresnel zone plate lens. Typical magnifications are of the order of × 1000. The sample is mounted on a rotary stage allowing the acquisition of different angular projections by rotating the sample around a vertical axis (*θ* rotation Fig. 1a, perpendicular to the X-rays).

The magnetic contrast is mathematically described via the dot product between the X-ray wave vector and the magnetization vector. The magnetic sensitivity via the XMCD effect allows to probe the magnetization components laying in a plane perpendicular to the rotation axis (in this configuration, *m*_*x*_ and *m*_*z*_)^26^. Hence, for getting information about the three components of the magnetization, a second tilt series is needed. The second tilt series is recorded after rotating the sample around the X-ray beam axis by ~ 90° [*φ* rotation Fig. 1a], thus in the new configuration the previously invisible component will be revealed, i.e., both *m*_*y*_ and *m*_*z*_ in this case. Experimental transmittance images for tilt series 1 (*φ * = 0°) and tilt series 2 (*φ*  = 75.2°) obtained from one of the Py microstructures (rectangle of 2 μm  × 1.3 μm) are presented in Fig. 1b. These are recorded at the Fe L_3_ edge (706.8 eV) with positive and negative circular polarizations under different projection angles ($\theta$). The closure domain structure of a diamond state is observed and the dot product nature of the XMCD contrast is clearly exemplified: although both tilt series are sensitive to the out-of-plane component of the magnetization equally, tilt series 1 (rotation axis parallel to the Y axis) allows to probe the in-plane magnetization mostly parallel to the long axis of the rectangle while in tilt series 2, the in-plane component probed is mostly parallel to the short axis of the structure. The change in polarization from circular positive (${C}_{+}$) to negative (${C}_{-}$) gives rise to the opposite contrast for the same magnetic configuration. Symmetric *θ* angles ($\theta =0^\circ$ is defined as colinear X ray and normal to the sample surface) for the same polarization, also give opposite in-plane contrasts. A double-tilt dataset (tilt series 1 and 2), with two polarizations, has been recorded from − 52$^\circ$ to 52$^\circ$ in steps of 8$^\circ$, including the angular projection at $\theta =0^\circ$, for three different rectangular structures.

## Methodogy

**Figure 2:**
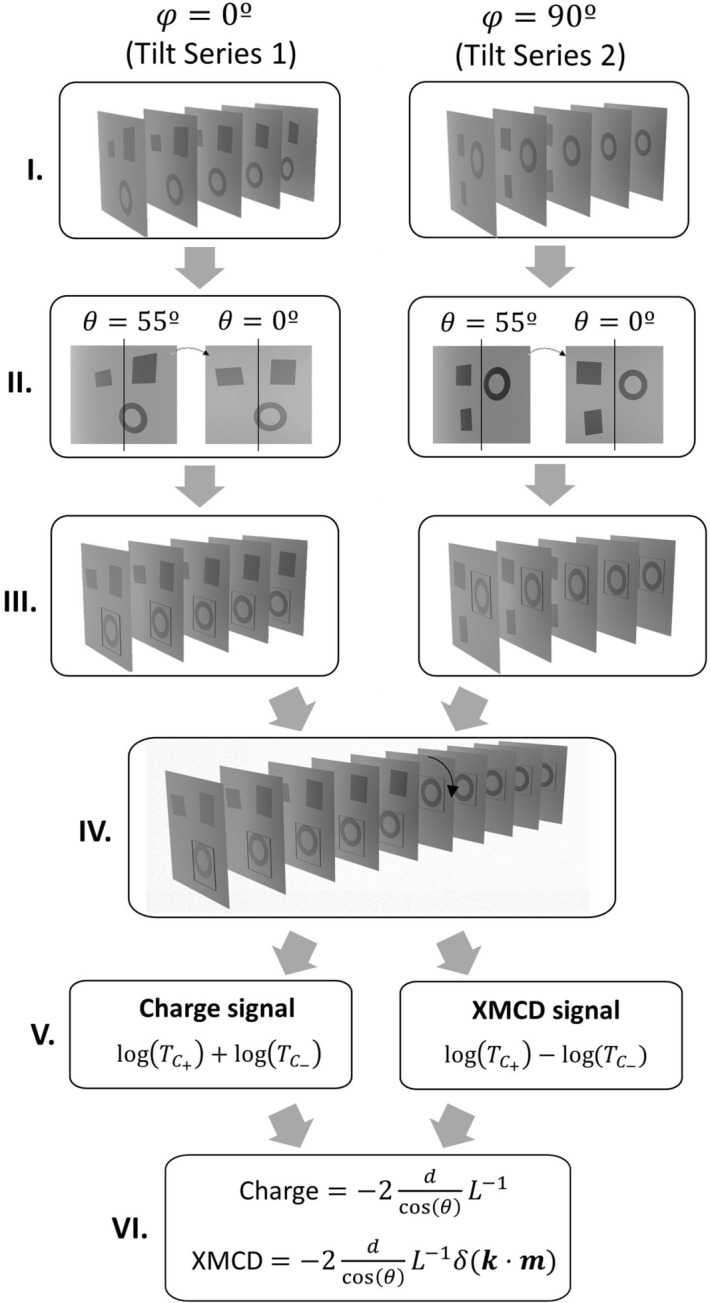
Flowchart for reconstructing the magnetic configuration from X-ray transmission measurements. I. Acquisition of two tilt series with $\varphi =0^\circ$ and $\varphi \approx 90^\circ$; II. Cosine stretching correction; III. Individual tilt series alignment; IV. Final dataset creation by the alignment and merging of both tilt series; V. Charge and magnetic contributions separation; VI. Magnetization reconstruction by Beer-Lambert equation fitting.

The process followed in this work to reconstruct the 3D magnetization of quasi-2D samples is outlined in the flow-diagram of Fig. 2. A more detailed discussion can be found in the Supporting Information S1. The first step (I) is to acquire the angular projections, namely, tilt series 1 (*φ*  = 0°) and tilt series 2 (*φ* ≈ 90°), with positive and negative circular polarizations as a function of the *θ* rotation angle. The range of this angle is usually limited to *− 70*° to + *70*° due to the closeness of the sample holder and zone plate as well as restrictions in the sample^27^. The second step (II) is to correct the cosine stretching by applying a scale factor of $1/cos\theta$ in the horizontal axis to both tilt series^28^. This effect leads to the decrease of the projected sample horizontal length as the tilt angle $\theta$ increases.

Due to the movement of the sample around the tilt axis, there is usually a small translation mismatch, both in the horizontal and vertical directions, between consecutive images. The following step (III) is to align the images to compensate this error for each tilt series. The angular projections must be aligned to a common reference system by correcting the cosine stretching, displacements and rotations. To avoid problems in the alignment since the magnetic contrast in the images depends on the *θ* angle, we generate and align masks with a regular step gradient descent algorithm. These masks saturate the magnetic part of the images allowing us to obtain the sample geometry avoiding any interference from the magnetic signal. As reference for the alignment algorithm, we choose the mask obtained at $\theta =0^\circ$. Then, the algorithm performs shifts, rotations, shears and/or scaling to match each mask with the reference one^29^. The transformation matrix obtained from the mask alignment, is applied to the original images to line up all the angular projections. This also allow us to compute the real $\theta$ angles for each angular projection. Finally, an intensity correction is applied between angular projections with the same tilt angles to compensate the unavoidable slightly different degree of polarization between circular right and left light.

After both tilt series 1 and 2 are separately lined up, we align the two series together to have the complete corrected dataset (IV).

In the fifth step (V), we take advantage of the XMCD effect to obtain the magnetic and charge contrast of the angular projections^26^. The transmittance *T*, which is the ratio between the transmitted and incident intensities of the X-ray beam, can be related to the magnetization vector in the sample by the Beer-Lambert equation, where vectors are represented with bold letters:

1 $${\text{T}} = \exp \left\{ { - \int {{\text{L}}\left( {\text{t}} \right)^{ - 1} (1 + \delta ({\text{t}}) ({\varvec{k}} \cdot {\varvec{m}}(t))) {\text{dt}}} } \right\}$$

*L*(*t*)^−1^ is the inverse of the attenuation length for X-rays, δ(*t*) is the dichroic coefficient for the XMCD in the magnetic material, ***k*** is the X-ray wavevector, ***m*** is the reduced magnetization vector (***m*** = ***M***/*M*_*s*_, with ***M*** the magnetization vector and *M*_*s*_ the saturation magnetization) and *dt* is the elementary path along the X-ray trajectory spanned by the line integral. By following the steps indicated in detail in^21^, we perform the addition and subtraction of the logarithms for the transmittances for opposite circular polarizations, obtaining the non-magnetic and magnetic contributions, respectively.

2a $$log\left({{T}_{C}}_{+ }\right)+ log\left({T}_{{C}_{-}} \right)= -2 \, \int L{\left(t\right)}^{-1} dt$$

2b $$log\left({{T}_{C}}_{+ }\right)- log\left({{T}_{C}}_{- }\right)= -2\int L{\left(t\right)}^{-1} \delta \left(t\right)\left({\varvec{k}} \cdot {\varvec{m}}\left(t\right)\right)dt$$

${{T}_{C}}_{+}$, ${{T}_{C}}_{-}$ represents the transmittance with circular right and left polarization respectively. The first equation (Eq. 2a) represents the non-magnetic part or charge, and the second equation (Eq. 2b) is related to the magnetic contribution or XMCD. By exploiting the fact that the thickness of the sample is below the typical through-thickness vector tomography resolution (~ 60 nm^14^), it is possible to reduce the reconstruction volume dimensionality to two dimensions. Considering the change in the effective thickness of the sample while rotating, these equations can be particularized as follows:

3a $$log\left({{T}_{C}}_{+}\right)+ log\left({T}_{{C}_{-}}\right)= -2\frac{d}{\mathit{cos}\left(\theta \right)} {L}^{-1}$$

3b $$log({{T}_{C}}_{+} ) - log({{T}_{C}}_{-} ) = -2\frac{d}{\mathit{cos}\left(\theta \right)} {L}^{-1} \delta ({\varvec{k}}\cdot {\varvec{m}})$$

where $d$ is the sample thickness and $d/\mathrm{cos}(\theta )$ is the effective sample thickness which depends on $\theta$. Finally, the reconstruction step is performed (VI), which consists of fitting the particularized Beer-Lambert equations to the experimental data. In the first step of the reconstruction, we compute the attenuation length, which is a scalar field, by a linear fitting of Eq. (3a). Then, we solve Eq. (3b) to obtain the reconstructed magnetization vector field by applying the Levenberg–Marquardt Algorithm which combines Taylor series and steepest-descent methods to ensure fast convergence^30–32^. In this step we use the previously computed attenuation length as a parameter.

## Reconstruction of thin films magnetization

After fitting the Beer–Lambert equation, the 3D magnetic configuration at each pixel is reconstructed for the three analyzed microstructures. In order to achieve a quantitative reconstruction, the dichroic factor $\delta$ and the attenuation length $L$ must be determined first. The dichroic factor is obtained from the transmission images in a Py micro-ring structure with known magnetization configuration (details in Supporting Information S1). We have determined $\delta =0.2602\pm 0.012$, which was used for all the reconstructions. From fitting Eq. (3a) we have obtained the attenuation length for the three microstructures (further details available in Supporting Information S1).

**Figure 3:**
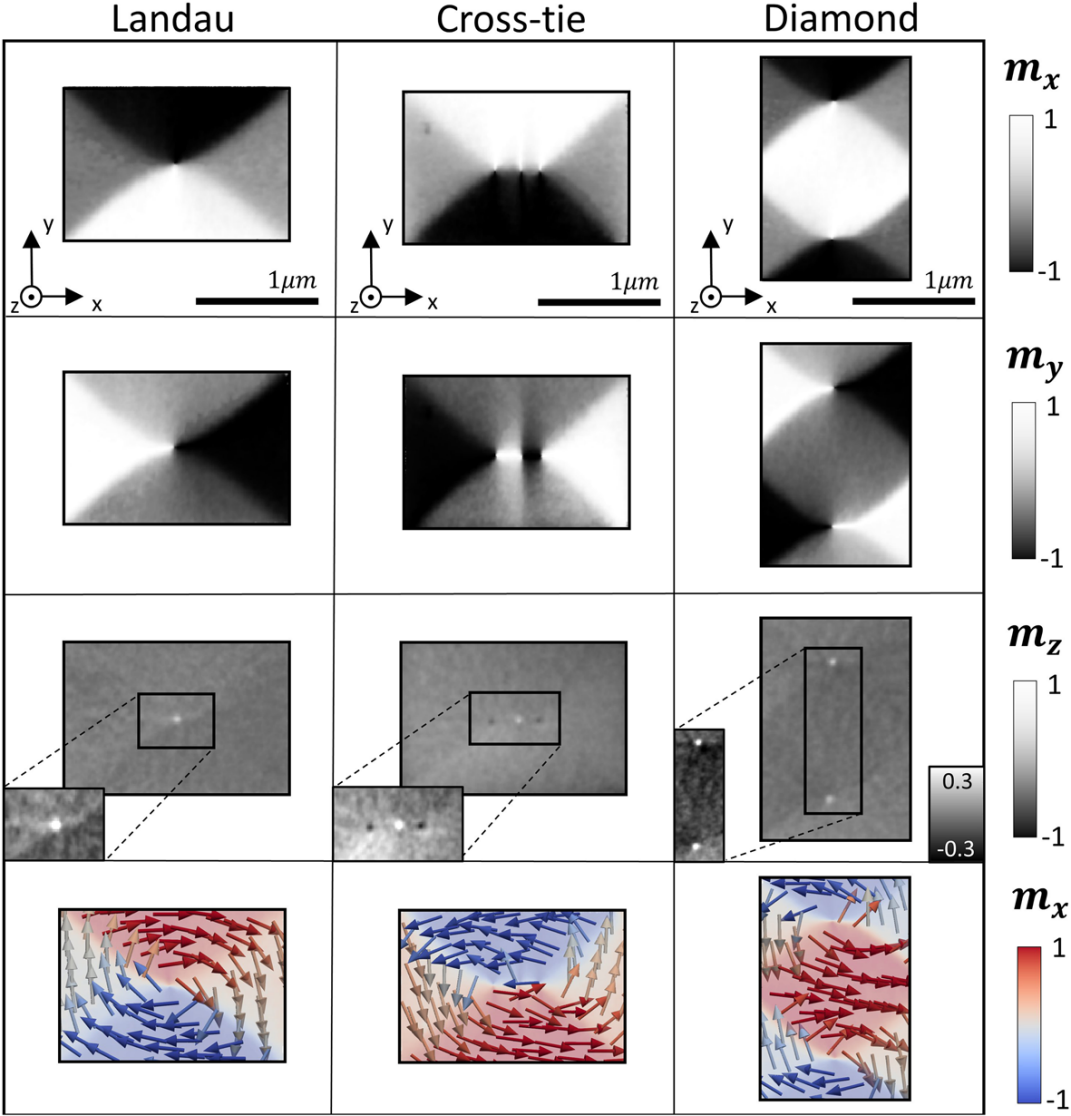
Three-dimensional reconstruction of Py films of 40 nm thick with 30 angular projections. Each row represents a component of the magnetization ${m}_{x}$, ${m}_{y}$, ${m}_{z}$. Each column refers to a different Py microstructure with different magnetization configuration: Landau vortex, Landau cross-tie, Diamond state. The insets in the third row present a magnified view of the vortices with the color scale adapted to show their polarity. Fourth row presents the vector representation of the reconstructed magnetization states.

Figure 3 shows the magnetic configuration of each Py microstructure with 30 angular projections. The first row represents the horizontal component of the magnetization ${m}_{x}$, while the second and third correspond to the vertical component ${m}_{y}$ and the perpendicular one ${m}_{z}$, respectively. Each column shows a different structure. The first structure (first column of Fig. 3) presents a Landau pattern with 90° Néel domain walls separating the triangular domains and a 180° wall close to the vortex in the middle of the rectangle. The second microstructure (second column of Fig. 3) shows another Landau configuration but containing a cross-tie wall separating the ${m}_{x}$ domains. This central cross-tie wall consists of two vortices with the same circulation and polarities leading to the formation of an antivortex in between them. In the ${m}_{z}$ component, opposite polarities for the vortices and the antivortex are observed. The last structure analyzed (third column of Fig. 3) presents a clear diamond state configuration formed by two vortices with different circulation but the same polarization. The lateral resolution of the reconstruction has been estimated to be around 50 nm by direct comparison with micromagnetically simulated data (further details in the Supporting Information S1). These results show the effectiveness of the algorithm in reconstructing the magnetization. Reducing the dimensionality from 3D to a quasi-2D system allows to drastically decrease the number of angular projections needed with respect to conventional magnetic vector tomography of 3D structures. Specifically, for the 30 angular projections recorded, the total acquisition time was around 3 h corresponding to an effective speed up in acquisition time of ~  × 10 for quasi-2D systems when compared with the conventional approach.

Finally, to further analyze the potential of the presented method, we have tested the effect of reducing the number of angular projections to 6 and performed different reconstructions. We have computed their error compared to the one calculated with the entire dataset, i.e., 30 angular projections. To achieve this, we have reduced tilt series 1 and 2 to three angular projections each. For both tilt series, the angular projection of *θ*  = 0° is always included and the other two angular projections correspond to nominally symmetric positive and negative *θ *measurements (i.e. − *θ*°, 0°, *θ*°). The minimum tilt angle necessary to be able to properly characterize the in-plane magnetization components of the system, is studied as a function of the angular separation (angular span) between opposite $\theta$ measurements. The reconstructed magnetization configurations with the reduced dataset for the microstructures can be found in the Supporting Information S1 for comparison.

As error metric we have calculated the normalized root mean square error (NRMSE) between reconstructed and experimental data as follows,

4 $$NRMSE=\frac{1}{\left({X}_{max}-{X}_{min}\right)}{ \left[\frac{1}{N}\sum_{i=1}^{N}{\left(X-{X}_{p}\right)}^{2} \right]}^{1/2}$$

where ${X}_{p}$ represents the XMCD images obtained from projecting the reconstructed magnetization, ${X}_{max}$ and ${X}_{min}$ are the maximum and minimum values of these projected images, $X$ are the experimental XMCD data and *N* is the total number of reconstructed pixels.

**Figure 4:**
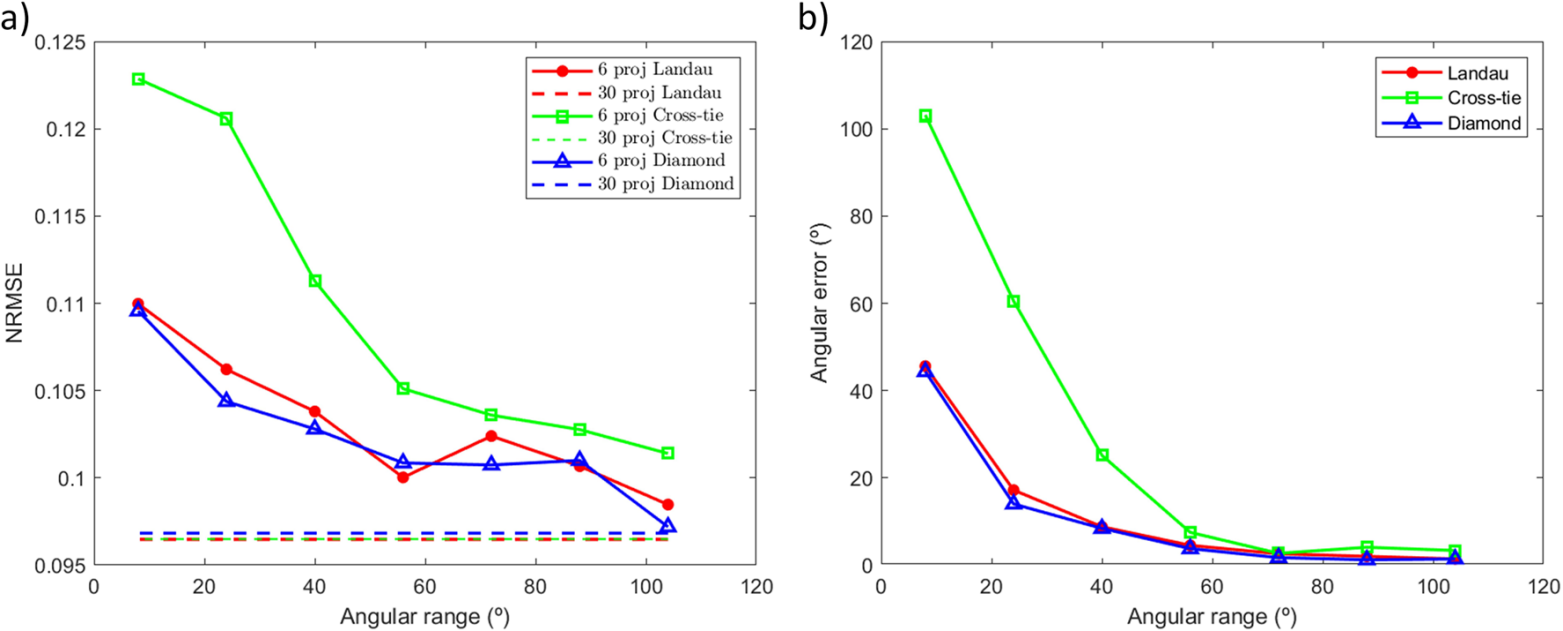
Errors for the reconstructed magnetic configurations as a function of the acquisition angular range. (**a**) Normalized root mean square error. The solid line shows the NRMSE when 6 angular projections are used in the reconstruction. The dashed line shows the NRMSE of the reconstructions with the whole dataset (30 angular projections). (**b**) Value of the average angular difference between the magnetizations reconstructed with all angular projections and with the reduced dataset (6 angular projections).

Figure 4a shows the NRMSE of the reconstructions obtained with 6 angular projections (3 from each tilt series) as a function of the angular separation between opposite *θ* values (solid lines). The dashed lines correspond to the error of the reconstruction with the complete dataset (30 angular projections from $-52^\circ$ to $52^\circ$). The three structures present similar trends: for smaller angular ranges, there is a larger error since we are sensitive mainly to the out-of-plane component of the magnetization while there is uncertainty in the determination of the in-plane components. For these reduced datasets of 6 angular projections, results show that above $50^\circ$ in the angular span, there is no large improvement in the error, it fluctuates ~ 4% around that value. For the highest angular span, the error is the closest to the base level associated to the complete dataset (30 angular projections), having relative differences of 1% for the diamond and Landau configurations and around 5% for the cross-tie. The reconstruction with the complete dataset considers all the angular projections explaining why its NRMSE level is lower.

Moreover, we have analyzed the magnetization vector angular error between the reconstructed magnetizations coming from the reduced and full datasets (Fig. 4b). A similar behavior to the NRMSE values is seen: the error reduces when the angular range increases, in agreement with the dot product nature of the XMCD contrast. Angular mismatches of less than $10^\circ$ between full and reduced datasets can be obtained if the angular span is larger than $50^\circ$. Angular errors of the same order have been identified in similar methodologies^22^.

Although a larger number of angular projections allows to obtain better results as indicated in Fig. 4a (dashed lines), it is possible to get qualitative information about the 3D magnetization configuration of quasi-2D systems by recording only 6 angular projections which implies even faster acquisition times, of the order of 15 min. The vector characterization approach proposed in this work increases thus the throughput of analyzed samples/states in quasi-2D geometries with respect to conventional vector tomography from ~ 10 (30 angular projections) to ~ 100 (6 angular projections) times, depending on the accuracy level required for the characterization.

## Conclusions

In this article, we have implemented a novel method to reconstruct the 3D magnetization vector field in quasi-2D materials and multilayers using soft X-ray transmission tomography. This method takes advantage of the reduced thickness of the sample compared with the axial resolution of the tomography setup to reduce the reconstruction volume to 2D, reducing the number of angular projections required for an accurate vector reconstruction (from few hundreds to 30 or 6 in this case).

We have recorded a complete double-tilt dataset composed of 30 angular projections (3 h acquisition time) revealing different 3D magnetization configurations present in three 40 nm thick Py microstructures: two Landau patterns, one mediated by a vortex and another by a cross-tie configuration, and a diamond state. The estimated lateral resolution is of 50 nm. By splitting the complete dataset into bunches of 6 angular projections, the accuracy of the reduced reconstructions has been analyzed as a function of the angular range employed for the characterization. Results show that a $50^\circ$ angular range is sufficient to reconstruct the magnetization vector field within the microstructures with good quality. The increase in the number of angular projections improves the results by increasing the signal to noise ratio and the amount of information taken into account for the reconstruction. However, with 6 angular projections it is possible to obtain qualitative reconstructions in less than 15 min of acquisition time in this system. Thus, the presented method is useful for performing reconstructions of the 3D magnetization vector field at the nanoscale for quasi-2D magnetic systems with speed ups in acquisition time around × 10– × 100 when compared with standard magnetic vector tomography experiments. Although the approach does not have the volume reconstruction capabilities of conventional tomography, it presents great potential for the Spintronics, Nanomagnetism and 2D-van der Waals magnetism communities where the quasi-2D geometrical constrain is usually fulfilled.

## Methods

### Sample preparation

First, a Si_3_N_4_ membrane (Ted Pella, 21501-10) with 50 nm thickness was spin-coated with the resist PMMA950K A4. Second, the ring and rectangle patterns were defined on the resine by conventional e-beam litography at 10 kV and with an exposition dose of 100 μC/cm^2^. After the development of the resist, a 40 nm layer of Permalloy (Ni_80_Fe_20_) was deposited on top of by DC magnetron sputtering with an Ar working pressure of 10^–3^ mbar. At last, the shapes were obtained by a lift-off process.

### Microscope

The soft X-ray transmission microscope of the MISTRAL beamline at ALBA synchrotron is installed on a bending magnet beamline equipped with a variable-line-spacing grating monochromator and focusing mirror optics. Circular right and left polarized radiation were obtained by modifying the orbit of the electrons to have a descending or ascending trajectory in the storage ring. A bounce glass capillary focused the photons into the sample. The microscope was operated with a magnification × 1300. The transmitted image had a 10.1 nm effective pixel size.

### Data acquisition

The experiment was conducted at the full field transmission soft X-ray microscope of the MISTRAL beamline at the ALBA Synchrotron. In this experiment, the XMCD was probed at the Fe L_3_ edge with a photon energy of 706.8 eV.

Series of angular images were acquired with different parameters due to the different degree of polarization. For circular right polarization the exposure time was 4 s and the number of images acquire at each angle is 20 to improve SNR. For left polarization, 24 projections were taken with 3 s of exposure each.

Two tilt series were acquired at $\varphi =0^\circ$ and $\varphi = 75.2^\circ$. Each one was measured starting from $\theta = -52^\circ$ to $\theta = 52^\circ$ in steps of 8° including the angular projection at 0° with both polarizations. Flatfields were collected at the beginning, middle and end of each tilt series and for each polarization with the same acquisition parameters.

## Supplementary Information

Supplementary Information.

## Data Availability

The datasets generated and analysed during the current study are available in the institutional repository of Universidad de Oviedo https://digibuo.uniovi.es/dspace/handle/10651/55750.
