# Supplementary material for: A fast magnetic vector characterization method for quasi two-dimensional systems and heterostructures
Source: Sci Rep. 2023 Jun 14;13:9639. doi: 10.1038/s41598-023-36803-z (PMC10267128; doi:10.1038/s41598-023-36803-z)
Supplement: Supplementary file 1 — Supplementary Information. [file 41598_2023_36803_MOESM1_ESM.pdf]

Supplementary Information of: A fast magnetic vector characterization method for quasi two-dimensional systems and heterostructures.

A.E. Herguedas-Alonso, L. Aballe, J. Fullerton, M. Vélez, J.I. Martín, A. Sorrentino, E. Pereiro, S. Ferrer, C. Quirós and A. Hierro-Rodriguez

Acquisition details and image preprocessing.

The transmittance images have been recorded in the MISTRAL full-field TXM at the ALBA synchrotron using circular right (C_+) and left (C_-) photon polarizations. The measurements have been performed at the Fe L_3 edge. Different projections of the sample with respect to the X-ray beam have been recorded with a magnification of x1300. In order to compute the transmittance for each projection, flat fields images (FF) are used for normalization. During the acquisition of the FF, the sample is removed from the X-ray path, thus, obtaining a direct measurement of the incident photon intensity.

In magnetic vector tomography, it is exploited the XMCD effect which allows to obtain the components of the magnetization parallel to the X-ray beam. So, for getting information about the three components of the magnetization, two tilt series are acquired for $\varphi = 0°$ and $\varphi = 90°$ nominally due to the fact that the X-ray beam is perpendicular to the rotation axis. For each φ configuration, projections have been recorded for tilt angles from $\theta = -52°$ to $\theta = 52°$ in steps of 8° including the measurement at $\theta = 0°$. FFs have been acquired at the begging, middle and ending of each tilt series or when changing the polarization in order to minimize the effects of drifts in the illumination of the microscope.

For each tilt angle, several projections are acquired in order to improve the signal to noise ratio. Each individual image has been converted to counts per second (3 - 4 seconds exposure time) and applied a correlation threshold to discard bad quality images from the stack. FFs are applied to compute the transmittance and the images have been aligned and averaged. Therefore, the dataset consists of the images obtained at each tilt angle (θ) with two polarizations (C_+, C_-) for two rotations of the sample (φ).

The next step is to align all the angular projections. First, we have generated a mask for each angular projection to perform the alignment. These masks allow to obtain the geometry of the sample and remove any magnetic signal. For each tilt series, the reference mask has been chosen by selecting the image with higher transmittance, corresponding to $\theta = 0°$. The alignment algorithm finds the geometrical transformation that aligns each mask with the reference one by minimizing the mean square error of the difference. As an initial transformation, the center of mass of each mask has been computed. The resulting affine matrix corrects the translation mismatches and the effect known as cosine stretching, in which the effective sample length decreases a θ increases. This allows to accurately obtain θ for each angular projection. Then, the transformation has been applied to the angular projections, so each tilt series is aligned separately to their specific reference. Due to the different degree of polarization between $C_+$and C_- because of the optics of the microscope, an intensity correction factor of around 1.2 has been applied in the images recorded with C_-. This factor has been obtained by comparing the

intensities of the angular projections with the same tilt angles but different polarizations. This allows to have the same magnetic contrast for both polarizations.

After performing the alignment for the tilt series separately, the alignment algorithm has been used again to join both tilt series. The transformation matrix allows to recover φ. Once the masks are properly aligned, the same affine transformations have been applied on the transmittance images.

Finally, as explained in [20], the non-magnetic and magnetic contributions are obtained by adding and subtracting the logarithms of the transmittance images with opposite polarizations before performing the reconstruction.

Dichroic coefficient.

To obtain a quantitative magnetic reconstruction it is important to determine the dichroic coefficient for the magnetic material. It represents the difference in the absorption of the material for circularly right or left polarized X-rays, giving the magnetic sensitivity of the dichroic phenomenon. This coefficient is usually characterized by conventional XMCD X-ray Absorption Spectroscopy measurements after saturating the sample along the X-ray direction. The last point is important as having the magnetization parallel to the X-ray beam simplifies the characterization of the dichroic coefficient. However, if a magnetic structure is created where the magnetization configuration is known, then it is possible to compute the dichroic coefficient directly without the necessity of applying external fields. Hence, we have created a 40 nm thick Py ring microstructure where the magnetization has all possible in-plane orientations due to the magnetic ring ground state. This allows us to have regions with the magnetization parallel and antiparallel to the photon's spin giving the maximum XMCD contrast.

The XMCD image of the Py micro-ring recorded for $\theta = 44°$ is shown in Fig. S1. The structure presents a ring state where the magnetization is rotating in-plane along the ring due to shape anisotropy, as the arrows in the XMCD image indicate. The dichroic coefficient is calculated from the L-edge transmittance spectrum of Fe shown in Fig. S2(a). The spectrum is measured with only with C_+ polarization. Red and green lines correspond to the two opposite zones of the micro-ring where the dot product between the magnetization and X-ray wave vector is maximum for this tilt angle (inset Fig. S2(a)). In the bottom row, it is plotted the asymmetry ratio (AR) in black, defined as:

$$AR = \frac{\log(T_1/T_2)}{\log(T_1 \cdot T_2)} \qquad \text{(S1)}$$

where T_1, T_2 are the maximum and minimum value of the transmittance images integrated in the green and red areas respectively. The AR signal shows the energy range where the absorption magnetic contrast is higher. Nevertheless, as the transmission spectrum is taken at $\theta = 45°$, the dichroic coefficient extracted needs to be corrected by a factor of $1/sin(\theta)$ in order to compensate for its underestimation due to the rotation of the structure with respect to the X-ray beam. The AR with and without the correction taken into account plotted in Fig. S2(b). The value of the AR at L_3 edge, allows to obtain a dichroic coefficient $\delta = 0.2602 \pm 0.012$.

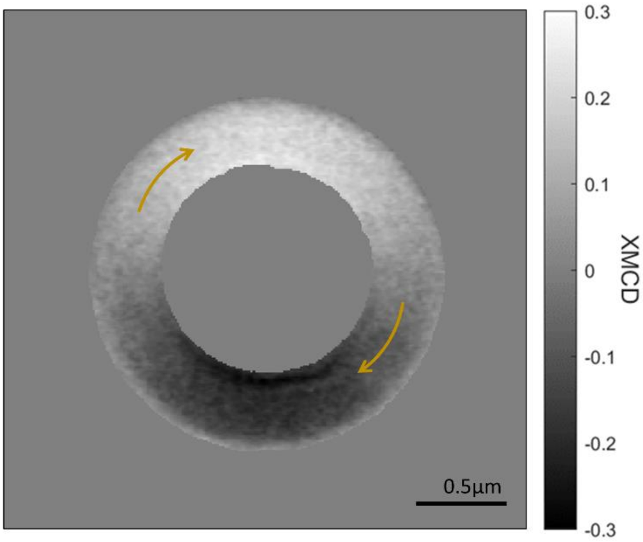

Figure S1. XMCD image of ring structure to obtain dichroic coefficient at $\theta = 44°$. The arrows indicate the direction of the magnetization.

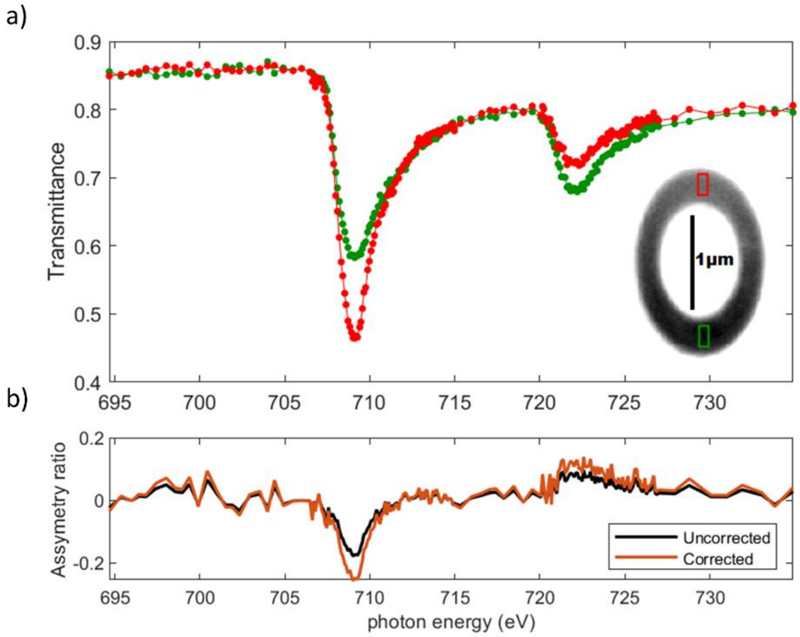

Figure S2. Top row shows the L-edge transmission spectra of Fe at $\theta = 45°$ for the regions with maximum (red) and minimum (green) values of magnetization. Bottom row shows the asymmetry ratio spectra from the transmission measurements and its value with the angle correction.

Attenuation length.

The attenuation length (L) is defined as the depth into the material measured along the surface normal where the intensity of X-rays falls a factor e of its value. It depends on electron density of the sample and on the energy of the photons. From the Beer-Lambert equation it is possible to recover this scalar coefficient by computing the addition of the logarithms of the transmittance for circular right and left polarizations.

$$log(T_{C_+}) + log(T_{C_-}) = -2 \int L(t)^{-1} \, dt \quad \text{(S2)}$$

where T_{C_+}, T_{C_-} represents the transmittance with circular right and left polarization respectively. In our case, the samples of Py were fabricated on top of Si_3N_4 membranes, therefore, our effective attenuation length is affected by both materials, L and L_{Si3N4}.If the reconstruction volume is reduced to two dimensions and considering the angle of rotation of the sample θ, which modifies the effective thickness, L has been obtained by fitting the following equation then:

$$log(T_{C_+}) + log(T_{C_-}) = -2\frac{1}{\cos(\theta)}(L\, d_{Py} + L_{Si3N4}d_{Si3N4}) \qquad \text{(S3)}$$

where $d_{Py} = 40\ nm$ is the nominal thickness of the Py deposited layer and $d_{Si3N4} = 50\ nm$ is the thickness of the Si_3N_4 membranes. The attenuation length of the membranes, L_{Si3N4}, is obtained by fitting Eq.(S4) with the transmission images where there is only the membrane.

$$log(T_{C_+}) + log(T_{C_-}) = -2\frac{1}{\cos(\theta)}L_{Si3N4}d_{Si3N4} \qquad \text{(S4)}$$

This yields a value of $L_{Si3N4} = (6.78 \pm 0.6) \cdot 10^{-7}\ m$. Results of the value for L are shown in Fig. S3 for the three microstructures analyzed.

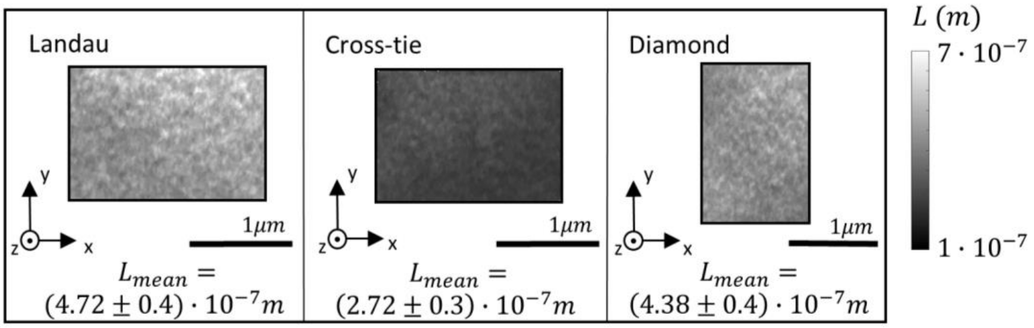

Figure S3. Reconstructed values for the attenuation length for the three microstructures. Below, it is shown the mean value.

Reconstructions with the reduced dataset.

With our algorithm, we have performed a series of reconstructions for the three Py microstructures with a reduced dataset of 6 angular projections for different values of the angular range. This angular range is defined as the difference in the θ value for the selected angular projections considered for the reconstruction. In Fig. S3, we show the reconstructions with the minimum and maximum value of the angular span and compare it to the one with the complete dataset (30 angular projections). When the angular range is minimum, there is uncertainty in the reconstruction of the in-plane components of the magnetization as can be seen from the large noise present in the reconstructions. The out-of-plane component is recovered since in this configuration the projections taken are the closest to the normal incidence (nominally $\theta = -8°, 0°, 8°$). On the other hand, the reconstruction from the largest angular range (nominally $\theta = -52°, 0°, 52°$) clearly shows well resolved in-plane magnetization components. The main difference between the latter reconstruction and the one computed with the complete dataset is related with a slight improvement of the signal to noise ratio in the last one.

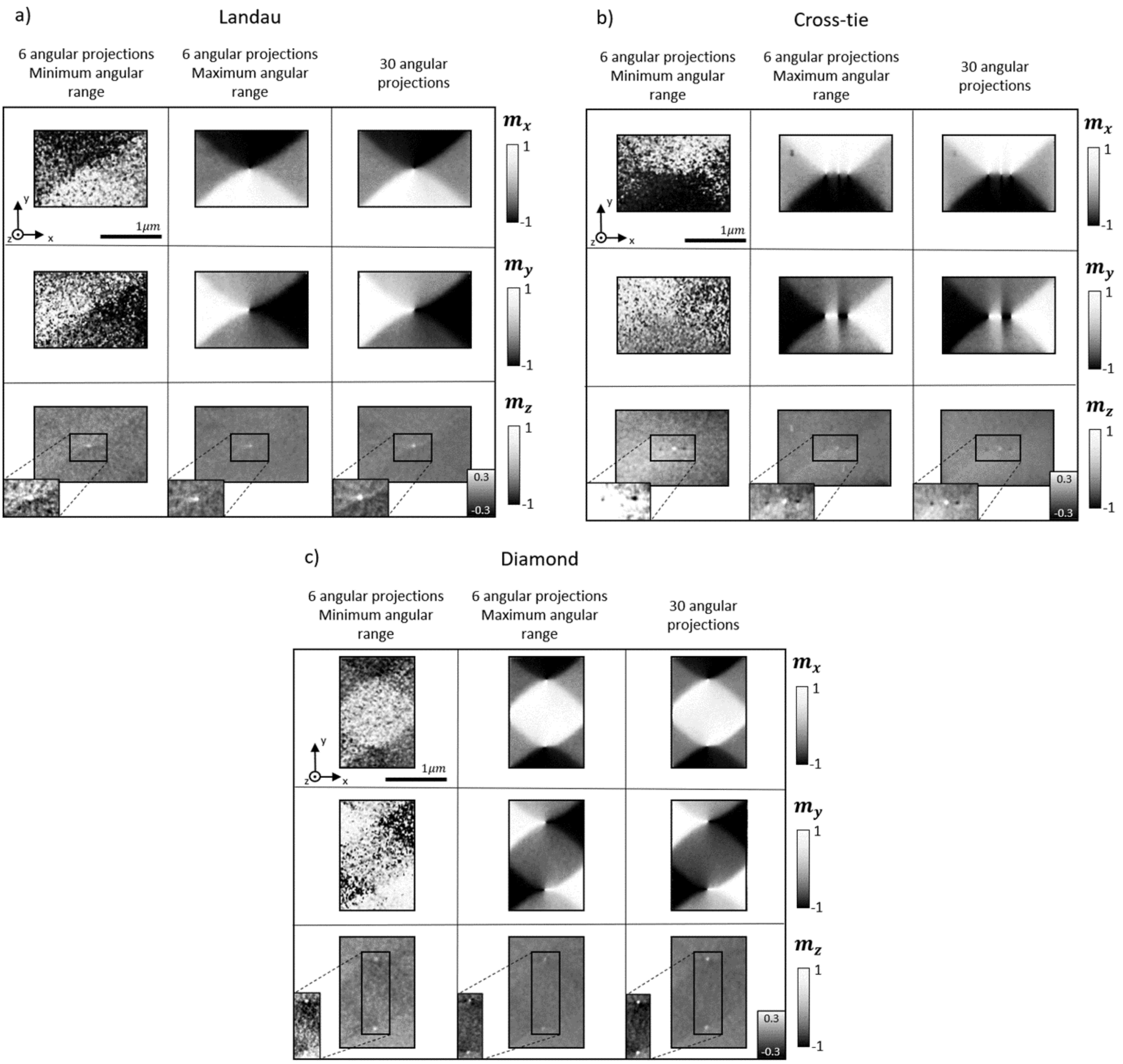

Figure S4. Reconstructed magnetization for Py microstructures with 40 nm thick with the reduced dataset (6 angular projections) at the minimum and maximum angular range and with the complete dataset (30 angular projections). a) Landau pattern; b) cross-tie configuration; c) diamond state.

Lateral resolution.

To estimate the lateral resolution, we have analyzed the reconstruction of the Landau vortex configuration. In this configuration, the x component of the magnetization near the vortex core presents a 180º domain wall. This domain wall is observed in the reconstruction as the result from the convolution between the true configuration of the magnetization and the resolution function of the reconstruction which also includes the microscope resolution. In order to estimate the reconstruction resolution, we have used a normalized Gaussian function of the form $f(x) = {}^{1}/_{\sigma\sqrt{2\pi}} \exp -(x^2/2\sigma^2)$, where the σ parameter is related with the full-width-

half-maximum value. A ground truth of the Landau vortex configuration is generated by a micromagnetic simulation using Mumax3 [1]. We have used a geometry consisting of a rectangular prism with the size of the sample (1750 nm x 1200 nm x 40 nm) with a discretization of 2.5 nm x 2.5 nm x 2.5 nm cell size. The used magnetic parameters are standard for Permalloy: saturation magnetization M_s = 800 kA m^{-1} and exchange stiffness A_{exch} = 1.3 x10^{-11} J m^{-1}. An initial magnetization configuration of a vortex with same polarity and circulation than in the reconstructed data has been relaxed leading to a Landau vortex state almost identical to the experimental one. Figures S5 (a) and (b) show the m_x magnetization for both the simulation and the experimental reconstruction respectively. Two magnetization profiles through the 180° domain wall have been taken at the same position from the simulated and experimental datasets and are indicated with red lines in (a) and (b). Figure S5(c) shows the reconstructed (blue) and simulated (red) profiles. To obtain the lateral resolution, we have fitted the convolution (green solid line in Fig.S5 (c)) of the simulation profile with the Gaussian function against the reconstructed data, optimizing for the σ parameter. The convolution corresponding to the best fit is shown with a green line in Fig S5(c), while the optimized Gaussian function is presented in Fig S5(d). The latter allows us to estimate a ~50 nm lateral resolution for the reconstruction from its full-width-half-maximum.

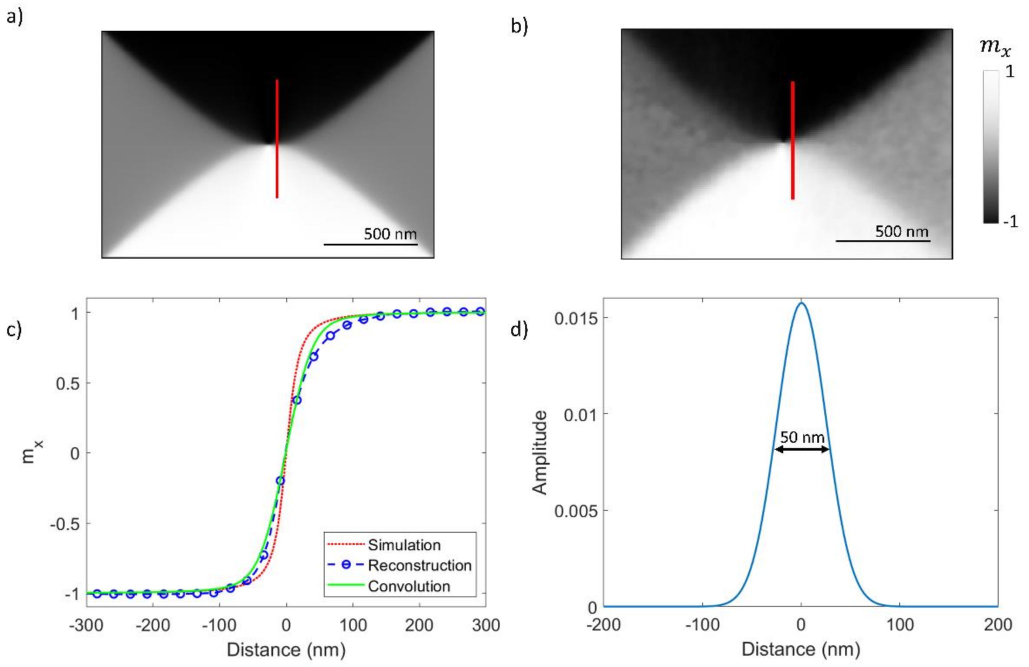

Figure S5. Mx component of the Landau vortex state obtained from simulation (a) and from the experimental reconstruction (b). c) Profiles of the simulation (red dotted line) and reconstruction (blue dashed line) of the domain wall. The best fit convolution between the Gaussian function and the simulation profile is represented with a green solid line. d) Gaussian function for the best fit case with a full-width-half-maximum of ~50 nm.

References.

[1] A. Vansteenkiste, J. Leliaert, M. Dvornik, M. Helsen, F. Garcia-Sanchez, B. Van Waeyenberge; The design and verification of MuMax3. *AIP Advances*, vol. 4, no. 10, 2014.
